# Alien vs. predator: bacterial challenge alters coral microbiomes unless controlled by *Halobacteriovorax* predators

**DOI:** 10.7717/peerj.3315

**Published:** 2017-05-31

**Authors:** Rory M. Welsh, Stephanie M. Rosales, Jesse R. Zaneveld, Jérôme P. Payet, Ryan McMinds, Steven L. Hubbs, Rebecca L. Vega Thurber

**Affiliations:** 1 Department of Microbiology, Oregon State University, Corvallis, OR, USA; 2 Department of Biological Sciences, University of Washington Bothell, Bothell, WA, USA

**Keywords:** BALOs, Halobacteriovorax, *Vibrio coralliilyticus*, Microbiome, Bacterial challenge

## Abstract

Coral microbiomes are known to play important roles in organismal health, response to environmental stress, and resistance to disease. The coral microbiome contains diverse assemblages of resident bacteria, ranging from defensive and metabolic symbionts to opportunistic bacteria that may turn harmful in compromised hosts. However, little is known about how these bacterial interactions influence the mechanism and controls of overall structure, stability, and function of the microbiome. We sought to test how coral microbiome dynamics were affected by interactions between two bacteria: *Vibrio coralliilyticus*, a known temperature-dependent pathogen of some corals, and *Halobacteriovorax*, a unique bacterial predator of *Vibrio* and other gram-negative bacteria. We challenged reef-building coral with *V. coralliilyticus* in the presence or absence of *Halobacteriovorax* predators, and monitored microbial community dynamics with 16S rRNA gene profiling time-series. *Vibrio coralliilyticus* inoculation increased the mean relative abundance of *Vibrios* by greater than 35% from the 4 to 8 hour time point, but not in the 24 & 32 hour time points. However, strong secondary effects of the *Vibrio* challenge were also observed for the rest of the microbiome such as increased richness (observed species), and reduced stability (increased beta-diversity). Moreover, after the transient increase in *Vibrios,* two lineages of bacteria (*Rhodobacterales* and *Cytophagales*) increased in coral tissues, suggesting that *V. coralliilyticus* challenge opens niche space for these known opportunists. *Rhodobacterales* increased from 6.99% (±0.05 SEM) to a maximum mean relative abundance of 48.75% (±0.14 SEM) in the final time point and *Cytophagales* from <0.001% to 3.656%. *Halobacteriovorax* predators are commonly present at low-abundance on coral surfaces. Based on the keystone role of predators in many ecosystems, we hypothesized that *Halobacteriovorax* predators might help protect corals by consuming foreign or “alien” gram negative bacteria. *Halobacteriovorax* inoculation also altered the microbiome but to a lesser degree than *V. coralliilyticus*, and *Halobacteriovorax* were never detected after inoculation. Simultaneous challenge with both *V. coralliilyticus* and predatory *Halobacteriovorax* eliminated the increase in *V. coralliilyticus*, ameliorated changes to the rest of the coral microbiome, and prevented the secondary blooms of opportunistic *Rhodobacterales* and *Cytophagales* seen in the *V. coralliilyticus* challenge. These data suggest that, under certain circumstances, host-associated bacterial predators may mitigate the ability of other bacteria to destabilize the microbiome.

## Introduction

Coral reefs have experienced sharp declines in coral cover from environmental factors (De’ath et al., 2012), temperature induced bleaching (Fitt & Warner, 1995), and disease (Bourne et al., 2009; Burge et al., 2014), with some areas of the Caribbean experiencing as much as 80% coral loss over the past several decades (Gardner et al., 2003). While many studies have identified microbial consortia that increase in diseased corals (e.g., Gignoux-Wolfsohn & Vollmer, 2015), etiological agents are generally unknown for the majority of coral diseases (Mouchka, Hewson & Harvell, 2010) and others are defined in broad terms as polymicrobial disease (Cooney et al., 2002). *Vibrio coralliilyticus* is a well described model bacterium for the study of interactions between corals, the environment, and pathogenic bacteria (Ben-Haim et al., 2003). Several *V.* *coralliilyticus* virulence factors are temperature-dependent and upregulated above 27 °C (Kimes et al., 2012), and it has been suggested that host tissue invasion can only occur above this threshold (Vidal-Dupiol et al., 2011). Given the continuous rise in sea surface temperatures due to global climate change (Hoegh-Guldberg et al., 2007), and the projected increased variability of temperature extremes, it is likely that the incidence of infections by *V. coralliilyticus* and other temperature-dependent pathogens will increase (Maynard et al., 2015). Bacterial communities of diseased corals are also known to have large numbers of opportunistic pathogens and secondary colonizers (Gignoux-Wolfsohn & Vollmer, 2015). It has been hypothesized that the majority of coral disease may be the result of normally-benign coral microbionts that become opportunistic pathogens during physiological stress to the host (Lesser et al., 2007a). Thus, the linkages between infection by a primary foreign agents and secondary opportunistic infections remain an area of active exploration.

Corals also form mutualistic and commensal partnerships with diverse microorganisms, ranging from endosymbiotic photosynthetic dinoflagellates (*Symbiodinium* spp.), to consortia of archaea, fungi, and bacteria. Although the role of *Symbiodinium* in the coral holobiont is well studied, the exact roles of each member of the bacterial portion of the holobiont remains far from clear. Experiments and metagenomic analyses have provided some insights into the roles of individual members of the coral microbiome (e.g., Wegley et al., 2007). It has been suggested that some of these bacteria provide direct benefits to the coral host, such as nitrogen fixation by symbiotic *Cyanobacteria* in *Montastraea cavernosa* (Lesser et al., 2007b), or ammonia oxidation by archaea (Beman et al., 2007). Other bacteria, particularly those in the coral surface mucus layer, are thought to provide a first line of defense against potentially invading foreign bacteria. Mucosal bacteria are thought to protect the host by several mechanisms, including production of antibiotics (Ritchie, 2006), secretion of chemical compounds that inhibit pathogen metabolism (Rypien, Ward & Azam, 2010), or competition for necessary resources and niche space (Ritchie & Smith, 1997). Increasingly, viruses and phages are recognized as also playing a regulatory role in the holobiont by controlling microbial populations (Barr et al., 2013; Soffer, Zaneveld & Thurber, 2014; Nguyen-Kim et al., 2014).

We have recently described how the predatory bacteria *Halobacteriovorax*, also likely influences the diversity and dynamics of the microbial community in the coral surface mucus layer through consumption of a broad range of bacterial prey (Welsh et al., 2016). *Halobacteriovorax* spp. are small, highly motile predatory bacteria that exhibit a biphasic lifestyle and prey exclusively on gram negative bacteria, including known coral pathogens (Williams, Falkler & Shay, 1980; Welsh et al., 2016). *Halobacteriovorax* are the marine component of a group of delta-proteobacteria known as *Bdellovibrio* and like organisms (BALOs). In free-living attack phase, BALOs actively seek out prey in order to attach, burrow inside, and restructure their host cell into a rounded bdelloplast. This kills their prey and provides BALOs with an osmotically stable structure free from competition to utilize prey resources for growth and replication. A new generation of attack-phase predators then bursts forth from the bdelloplast to seek new hosts.

Bacterial predators in the coral microbiome could be a type of top-down control, that directly alters the structure and function of the coral microbiome as demonstrated in other aquatic systems by bacterivorous predators (see reviews by Jürgens & Matz, 2002; Pernthaler, 2005; Matz & Kjelleberg, 2005). For example, we highlighted potential interactions of *Halobacteriovorax* and other members of the coral holobiont using co-occurrence network analysis of an in-field experimental time series of three coral genera, across three years, several treatments, and range of temperature conditions. These networks showed that *Halobacteriovorax* are core members of the coral microbiome, present in >78% of samples from three coral genera *Porites, Agarica,* and *Siderastrea* (Welsh et al., 2016; Zaneveld et al., 2016). We also showed that isolated strains of coral-associated *Halobacteriovorax* prey upon known coral pathogens in cultured settings. Such antagonisms between predators and prey in the holobiont may have variable effects on the microbiome, such that they could be occlusive to pathogens or disruptive to the coral microbiome itself. Here we examine how a specific bacterial predator (*Halobacteriovorax*), a foreign bacterium (*V. coralliilyticus*), and a coral host (*M. cavernosa*) interact to affect the complex system of the coral microbiome in a laboratory-based system. While *V. coralliilyticus* is not associated with causing disease in *M. cavernosa*, our study still provides baseline experiments for predator and prey interactions on a coral host.

## Methods

### Bacterial strains, growth conditions, and prey range assays

*V. coralliilyticus* are known to be naturally abundant (Wilson et al., 2013) and infect corals (Ushijima et al., 2014; Tout et al., 2015). Although, *V. coralliilyticus* has not been shown to cause disease in the coral *M. cavernosa,* we believe that *V. coralliilyticus* serves as an interesting model to investigate the interactions and effects of bacterial predation on a host, given that it is a well understood bacterium and is consumed by our model predator, *Halobacteriovorax*, a member of the coral core microbiome (Welsh et al., 2016).

We first evaluated if *Vibrios* can be preyed upon by coral-associated *Halobacteriovorax*, by conducting a series of predation assays in liquid and solid media. Bacterial strains *Vibrio fortis* PA1 and *Vibrio coralliilyticus* ATCC BAA450 (Accession # KT626460 and AJ440005, respectively), were grown on Marine 2216 Agar (MA) overnight. A single colony was re-suspended in 50 mL Marine 2216 Broth (MB) in a 250 mL flask at 30 °C and 250 rpm overnight. Cultures were diluted 1:100 in fresh media and incubated until late exponential growth before use in any experiment.

Our predatory bacterial strain, *Halobacteriovorax* sp. PA1 (Accession # KR493097), was grown as previously reported (Welsh et al., 2016) in Pp20 media. Briefly, a single plaque from a double layer plate was resuspended in 3 mL of 10^9^ *V. fortis* cells in filtered seawater in 15 mL test tubes. The culture was incubated at 28 °C and shaking at 250 rpm overnight. A 1:100 dilution of the overnight culture was prepared by adding 0.5 mL of 0.45 µm filtered culture to 50 mL of 10^9^ *V. fortis* PA1 cells in filtered seawater. This new co-culture of predator and prey was grown in a 250 mL culture flask at 28 °C and 250 rpm and monitored until late exponential phase before use in any experiment.

**Figure 1:**
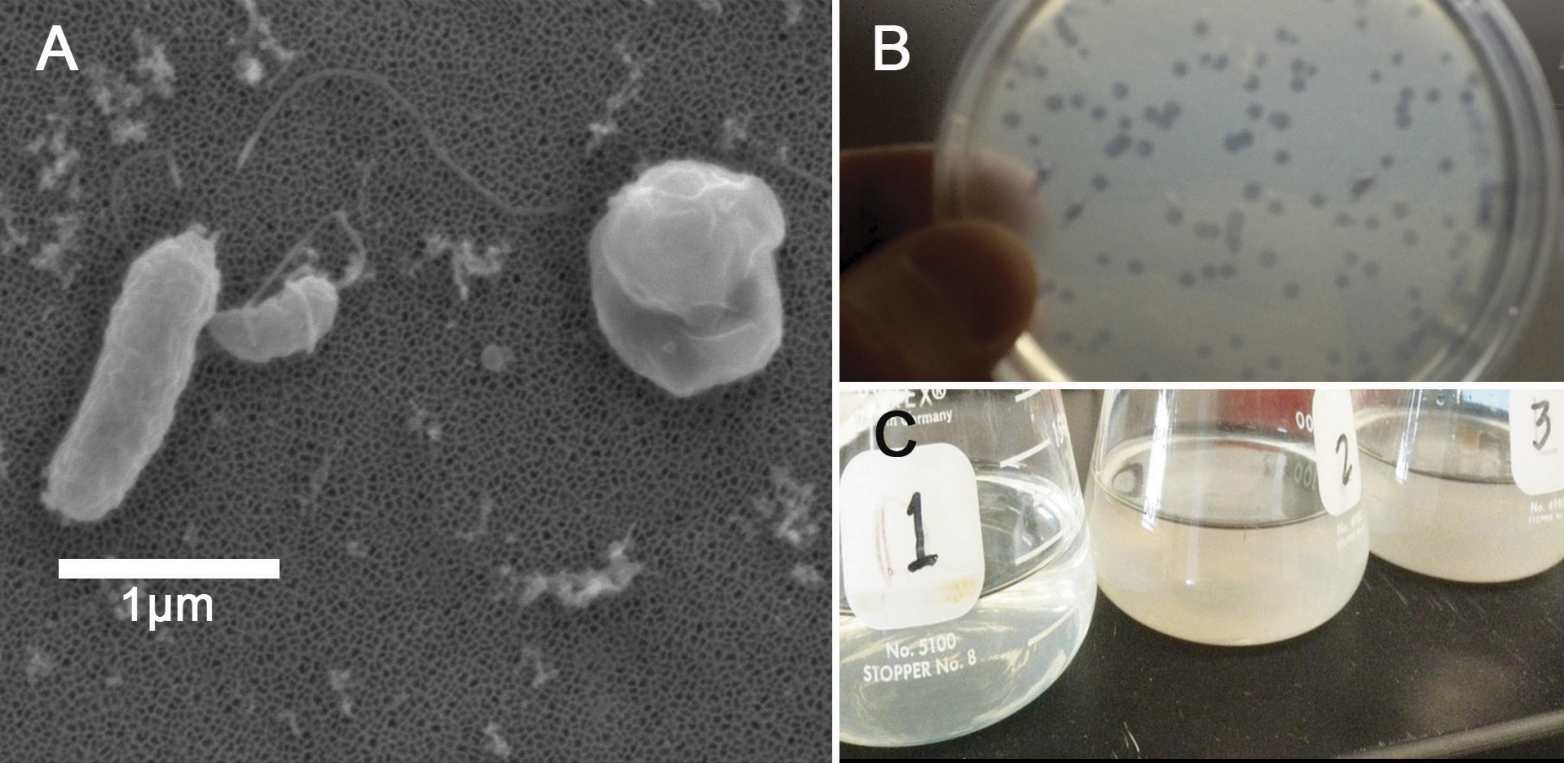
*Halobacteriovorax* predation of *Vibrio spp*. (A) Micrograph of the pathogen, *Vibrio coralliilyticus* BAA450 being attacked by *Halobacteriovorax* and rounded *V. coralliilyticus* bdelloplast (right) with *Halobacteriovorax* inside (B) double layer plate showing freshly lysed plaques on a lawn of *V. coralliilyticus* cells (C) Overnight liquid cultures of (1) a co-culture of *Halobacteriovorax* and *V. fortis,* (2) *V. fortis* and 0.2 µm filtrate from *Halobacteriovorax* culture, and (3) *V. fortis* alone.

The double layer technique assayed whether *Halobacteriovorax* sp. PA1 was capable of preying on various bacteria (Table 1). One milliliter of the potential prey bacteria suspension (containing 10^9^ cells/ml) and one milliliter of the appropriate predator dilution was mixed with 3 ml of molten agar (PP20 medium containing 1.1% Difco agar) held at 42 °C, for a final top layer agar concentration of 0.66%. The mixture was immediately spread over the surface of 1.8% agar PP20 Petri dish plates, and three replicates were plated for each predator–prey combination. Plaques were measured after 3–5 days of incubation (Fig. 1C).

**Table 1:** Bacterial predation assay results for several pathogenic *Vibrio* strains.

**Prey taxon (accession number)**	**50% killing rate in liquid media (hours)**	**Predation by Halobacteriovorax? (Double layer assay)**
*V. coralliilyticus BAA 450* (AJ440005)	19.00	Yes
*V. coralliilyticus RE 98* (CP009617)	24.16	Yes
*V. coralliilyticus RE22* (PRJNA168268)	No observable predation	No
*V. tubiashii* ATCC19106 (NZ_AFWI00000000.1)	No observable predation	No
*V. tubiashii* ATCC19109	13.84	Yes
*V. fortis PA1* (KT626460)	11.67	Yes
*V. cholerae N1696* (AE003853)	8.96	Yes
*V. cholerae S10* (accession)	12.76	Yes

Triplicate biological replicates for each vibrio prey species were grown overnight in marine broth at 28 °C and shaking at 250 rpm, transferred using a 1:100 ratio into fresh MB, and monitored until late exponential phase. Prey were washed three times in 0.2 µm filtered and autoclaved seawater (FSW) and resuspended to a concentration of 10^9^ cells/mL. Three biological replicates of overnight *V. fortis* and *Halobacteriovorax* sp. PA1 in FSW, which had lysed and cleared *V. fortis* prey, were 0.45 µm filtered to isolate predators. Filtered predators were then added to prey species at a 1:100 volume ratio. Predation was measured by OD_600_ values using a microplate reader (Infiniti M200; Tecan Group Ltd, Männedorf, Switzerland). Tecan OD values were reported without conversion to a 1-cm path length. *Halobacteriovorax* in attack phase do not significantly alter the absorbance reading of the prey at 600 nm due to their small cell size. Predation rates in the liquid co-culture assay were measured by the host cell density reduction compared to the predator-free controls. Based on our observed predation rates and the biological relevance of the strain, we chose to conduct our predator–prey addition experiment using *V. coralliilyticus* BAA 450 (accession # AJ440005).

### Collection and preparation of *Montastraea cavernosa*

*Montastraea cavernosa* was selected as a model for this work as it is both a common reef-building Caribbean coral and is susceptible to a variety of coral diseases (Sutherland & Ritchie, 2002; Goodbody-Gringley, Woollacott & Giribet, 2012). The 33 × 32 cm *M. cavernosa* colony used in the main experiment was obtained from the Florida Keys National Marine Sanctuary (#FKNMS-2010-123) from the Key West (FL, USA), and was maintained for 10 weeks in a shaded flow through raceway tank at the University of Miami Experimental Hatchery. This single *M. cavernosa* colony was split into 3.5 cm diameter cores with skeleton trimmed to ∼2 cm. Coral cores were transferred back into a common garden experimental aquaria that only contained the *M. cavernosa* cores and were allowed to acclimate for an additional four weeks where they demonstrated signs of growth including lateral tissue extension over exposed skeleton and feeding behavior during recovery. Just prior to being subjected to the various experimental treatments as described below, the *M. cavernosa* cores were then transferred to a second common garden at FIU which was composed of a recirculating seawater tank that only contained the *M. cavernosa* cores. All coral cores were distributed randomly into new 40L recirculating treatment tanks and only used once in the experiment. Seawater for the experiment was obtained from the University of Miami Experimental Hatchery (sand and UV-filtered seawater pumped in from Biscayne Bay).

### *Montastraea cavernosa* alien and predator additions

Bacterial challenges were conducted in sterile beakers with water temperatures held at 31 °C to induce *Vibrio coralliilyticus* virulence as previously described (Kimes et al., 2012). To further encourage *Vibrio coralliilyticus* to affect the host and/or microbiome all coral cores (including the controls and *Halobacteriovorax* challenges) were taken from common garden tank at time zero, scored with a file to mimic tissue damage, and inoculated in the beakers by transferring the sterile media (control), *V. coralliilyticus or Halobacteriovorax* cells using a sterile q-tip (Adwin Scientific, Schaumburg, IL, USA).

**Figure 2:**
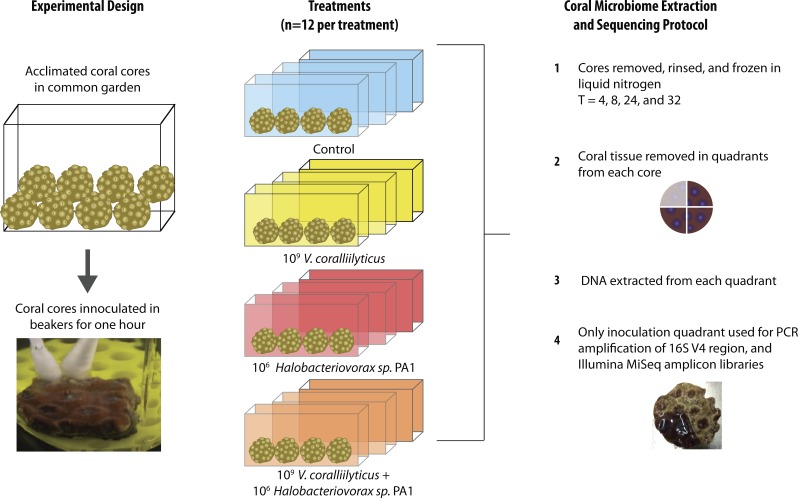
*Montastraea cavernosa* microbiome manipulation experimental design detailing collection and inoculation of coral cores, treatment tanks and replication, sample preservation, tissue removal, DNA extraction, and microbiome sample processing.

The 48 coral cores were divided into 4 treatments for the main experiment, providing 12 cores per treatment. These treatments were challenges of q-tips containing: (1) sterile media as a control, (2) a total of 10^9^ *Vibrio coralliilyticus,* (3) a total of 10^6^*Halobacteriovorax,* and (4) a total of 10^9^ *V. coralliilyticus* and 10^6^ *Halobacteriovorax*. Three replicate cores (one from each of the 3 replicate treatment tanks were collected at each of 4 time points per treatment (3 replicates × 4 timepoints = 12 per treatment; 12 × 4 treatments = 48 cores total)) (Fig. 2). Once challenged with the treatments, as described above, the fragments were placed in 40 liter tanks with natural seawater but constant recirculation, filtration, and temperature control for the 32 h experimental duration.

In each experimental challenge, late exponential phase bacteria cultures were pelleted, supernatant removed, and the cells washed three times with sterile artificial seawater (ASW) in 2 mL tubes by centrifugation at 10,000 × g for 10 min and gently re-suspension with sterile media. The late exponential *Halobacteriovorax* culture was passed through a 0.45 µm filter to remove prey and washed three times under the same condition as the *V. coralliilyticus* cells. The final cell pellet was resuspended in 100 µl of ASW and transferred using a dual q-tip approach (media control and media control, Vc and media control, Hbv and media control, and Vc and Hbv) to apply cells to freshly abraded corals in sterile beakers (Fig. 2). Q-tips were held on corals for 1 h in the inoculation beakers before transferring the challenged cores to recirculating seawater tanks. Laboratory experiments to quantify the number of cells that remain attached to the q-tip during transfer of the cell pellet were conducted by inoculating 1 ml of media for an hour, removing the q-tip, and performing direct counts of the ASW tubes using epifluorescent microscopy.

At each time point (*T* = 4, 8, 24, and 32 h) one coral fragment from each replicate tank of each treatment (*n* = 3 per time per treatment) was removed, photographed, placed in a Whirlpak (Nasco, Salida, CA, USA), flash frozen in liquid nitrogen, and transferred to −80 °C freezer for microbial DNA analysis.

### *Montastraea cavernosa* microbiome DNA extraction, sequencing, and quality control

From each core one quadrant of the coral tissue layer was removed using a dental tool and transferred into separate microcentrifuge tubes (4 per core) containing 500 µl of TES Buffer (10 mM Tris–HCl [pH 7.5], 1 mM EDTA, and 100 mM NaCl). A 1.5 mL microtube pestle was used to homogenize the tissue before adding 400 µl of TES buffer with lysozyme (Epicentre; final: 10 U µl^−1^), followed by incubation at 37 °C for 30 min. A 200 µl aliquot of homogenized sample was used for DNA extraction with the Power Soil DNA extraction kit (MoBio Laboratories, Carlsbad, CA, USA); the remainder was stored at −20 °C. Microbial amplicon libraries were generated using 515F and 806R primers to the V4 region of the 16S rRNA gene with Schloss sequencing adapters (Kozich et al., 2013). AccuStart II PCR ToughMix (Gaithersburg, MD, USA) and the following thermocycling conditions were used for amplification:1 cycle of 94 °C for 3 min; 35 cycles of 94 °C for 30 s, 50 °C for 30 s, and 72 °C for 60 s; and 1 cycle of 72 °C for 10 min were used for amplification. Each sample underwent triplicate reactions that were pooled and cleaned using the Promega Wizard SV Gel and PCR Clean-Up System (Madison, WI, USA). The samples were then quantified using a Qubit dsDNA HS kit (Invitrogen, OR, USA) before being pooled in an equimolar ratio. The amplicon purity and length was checked on an Agilent Bioanalyzer 2100 prior to sequencing on a MiSeq Illumina sequencing platform at the Oregon State University’s Center for Genome Research and Biocomputing (CGRB) Core Laboratories.

Quality control and selection of operational taxonomic units (OTUs) was performed using QIIME (v.1.8) (Caporaso et al., 2010). Sequences with quality scores less than a mean of 35 were removed. Sequences were clustered into (OTUs) at a 97% 16S rRNA gene identity threshold using USEARCH 6.1.54 (Edgar, 2010) and the subsampled open-reference OTU-picking protocol in QIIME v.1.8 (Rideout et al., 2014), using greengenes 13_8 as the reference (McDonald et al., 2012). Chimeric sequences were removed with QIIME’s wrapper of the UCHIME software (Edgar et al., 2011). Singleton OTUs were removed. The OTUs were assigned taxonomic classification using the QIIME wrapper to the UCLUST software package (Edgar, 2010). OTUs that were classified as chloroplast, eukaryotic or mitochondria were filtered out of the dataset.

### Statistical analysis

To avoid artifacts due to uneven sampling depth during comparisons of alpha and beta diversity, all samples were rarified (randomly subsampled) to equal sequencing depth. After quality control steps, the least sequenced sample had 11,716 reads; this value was thus chosen as the rarefaction depth. For alpha diversity (richness), total observed OTUs and Chao1 diversity statistics (Chao, 1984) were calculated in QIIME. The significance of differences in alpha diversity across treatments was determined in QIIME using nonparametric *t*-tests with 999 Monte Carlo permutations. For beta diversity analysis, weighted UniFrac distances (Lozupone & Knight, 2005) were calculated in QIIME. Distances within samples in each treatment category were summarized and tested for significance using Monte Carlo Permutation tests (make_distance boxplots.py, non-parametric *p*-value, *n* = 999 permutations). To account for multiple comparisons between treatments, Bonferroni-corrected *p*-values are reported for both alpha- and beta-diversity analyses in the text and figures.

To analyze how order-level taxa responded to bacterial challenge, a generalized linear model (GLM) was fitted with the R package DESeq2 (Love, Huber & Anders, 2014). The GLM design specified time point, treatment, and their interaction as factors. For this analysis, the order level OTU table was pre-filtered in QIIME where we excluded all taxa present in fewer than 6 samples. To test for the effect of treatment, a full model was compared to a reduced model fit using only time as a factor, and likelihood ratio tests (LRT) were performed to assess taxon (Table 2). Post hoc Wald tests were performed on the full model object to identify the specific treatments responsible for driving changes in these taxa. To control the rate of false positives due to multiple comparisons, differentially abundant taxa were identified as taxa with Benjamin-Hochberg FDR *q*-values less than 0.05.

**Table 2:** Bacterial taxa significantly altered by bacterial challenge treatment. Likelihood ratio test and post hoc Wald test statistics based on sequences derived from coral samples in microbiome manipulation experiment and Benjamini–Hochberg corrected *p*-values reported for five order level taxa (*α* ≤ 0.05 reported in bold).

	Likelihood ratio test for GLMs	Post hoc Wald test on individual treatment comparisons with Benjamini–Hochberg correction					
**Taxa significantly altered by treatment (order level)**	***P* value with Benjamini–Hochberg correction**	**Control vs. Hbv**	**Control vs. Vc**	**Control vs. Vc & Hbv**	**Hbv vs. Vc & Hbv**	**Vc vs. Vc & Hbv**	**Vc vs. Hbv**
Burkholderiales	**0.007**	0.864	0.558	0.210	0.093	**0.015**	0.850
Vibrionales	**0.011**	0.644	**2.08E–04**	0.829	0.429	**0.001**	**0.040**
Cytophagales	**0.014**	**7.21E–05**	**9.23E–05**	**0.002**	0.429	0.424	0.958
Alteromonadales	**0.036**	0.936	0.616	**0.014**	**0.020**	**4.46E-04**	0.835
Rhodobacterales	**0.045**	0.644	**0.001**	0.393	0.987	**0.025**	0.060

## Results

### *Halobacteriovorax sp.* PA1 can prey on multiple *Vibrio* species

Strain and species level differences in susceptibility to predation were detected among some of the Vibrio species (Table 1). For example, among the *V. coralliilyticus* strains, BAA 450 was most susceptible to predation in the liquid assay while strains RE22 and RE98 were less susceptible (Table 1). In the double layer plate assay, *Halobacteriovorax sp.* PA1 was capable of killing prey and forming plaques on all *Vibrio* spp*.* except *V. coralliilyticus* RE22 and *V. tubiashii* ATCC19106 (Table 1; Fig. 1C). *Halobacteriovorax* predation rates (50% killing) ranged from 8.96 h in *V. cholerae* N1696 to 24.16 h in *V. coralliilyticus* RE98. The prey rate for *V. coralliilyticus* BAA 450, a model coral pathogen, was 19.00 h.

### Vibrio coralliilyticus treated corals experienced altered coral microbiome *α*- and *β*-diversity

**Figure 3:**
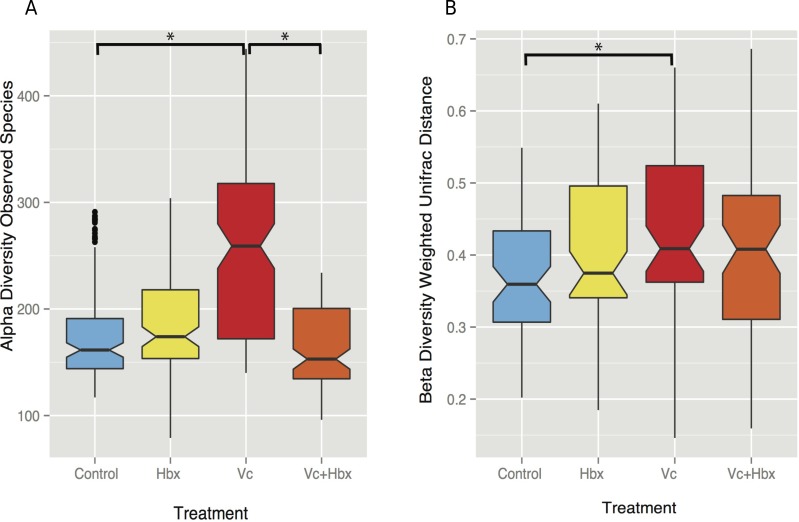
Impact of bacterial challenge on coral associated microbial diversity. (A) Mean alpha diversity (observed species) plotted for each treatment, and (B) mean beta diversity (Weighted UniFrac distance) by treatment. The asterisks indicate Bonferroni-corrected *p* values <0.05 for the nonparametric *t*-test between treatments. In both cases while addition of the pathogen alone increased diversity, predator addition counteracted this effect.

Given the observation that *Halobacteriovorax sp.* PA1 was capable of killing *V. coralliilyticus* BAA 450 in co-culture, we conducted an *in situ* challenge experiment that directly inoculated *M. cavernosa* corals with this foreign bacterium in the presence and absence of the predator (Fig. 2). Corals were inoculated using a swab transfer method and laboratory experiments indicate that an average of 6.02 × 10^8^ (±6.63 ×10^7^) total *V. coralliilyticus* cells were transferred using the swab transfer method (Table S1). No significant differences were observed in tissue loss or bleaching among the treatments at any of the time points (data not shown). We quantified relative microbial changes using 16S rRNA sequencing of corals in each treatment at 4, 8, 16, and 32 h post inoculation. After quality filtering of the experimental microbiomes, 4,464,765 reads remained with an average of 85,860 ± 112,003 reads per samples. After rarefaction the mean number of observed OTUs across all samples was 197 (Fig. 3A).

*Vibrio coralliilyticus* challenge increased microbial alpha diversity in the tissues of *M. cavernosa* corals. Corals challenged with *V. coralliilyticus* showed significantly increased richness relative to controls (*Vibrio* mean = 259.767 ± 14.196; control mean = 178.167 ±47.398) as measured by Chao1 and observed species diversity metrics (*p* = 0.048; Fig. 3A). However, when *M. cavernosa* samples were co-inoculated with both *V. coralliilyticus* and *Halobacteriovorax*, species richness returned to lower levels (mean = 163.058 ± 36.772) that were similar to control conditions, but distinct from the *V. coralliilyticus* treatment (*p* = 0.018; Fig. 3A). Changes in alpha diversity occurred early in the experiment, and normalized for all treatments, except the *V. coralliilyticus* treatment which gradually increased over the remaining time points by 48% (Table S2). No significant differences in evenness were observed between treatments. Further no significant differences in α-diversity were found between tanks or time points for either the Chao1 or observed species metrics.

Weighted UniFrac distances (*β*-diversity) were also significantly different between the control treatment and the *V. coralliilyticus* treatment (*p* = 0.012) (Fig. 3B). In a similar pattern to α-diversity, the *Halobacteriovorax* and *V. coralliilyticus* combination treatment returned *β*-diversity to control levels and were not significantly different than the other treatments (Fig. 3B). The *Halobacteriovorax* sp. PA1 alone did not significantly change *β*-diversity, and no significant differences were found between tanks or time points for *β*-diversity metrics.

### *Vibrio coralliilyticus* inoculation increases opportunists like *Rhodobacterales* and *Cytophagales* unless controlled by predators

**Figure 4:**
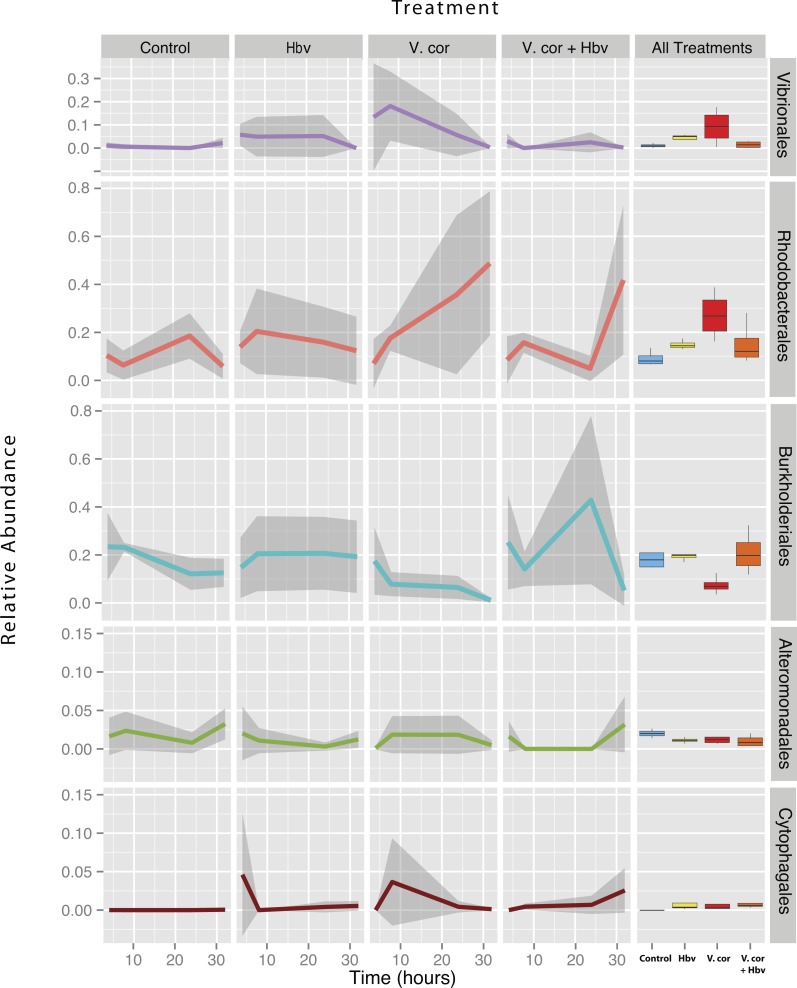
Relative abundance of all taxa found to be differentially present under the four bacterial challenges. Colored lines denote mean relative abundance for each time point with grey transparent shading indicating the standard deviation. Boxplots show the mean relative abundance averaged across all time points for each treatment.

Despite the inoculations lack of any visual signs of pathogenesis, we still found significant differences in the relative abundance of 16S rRNA genes to both Vibrio and other taxa associated sequences in the tissue microbiomes of the challenged corals (Fig. 4). To test if inoculation treatments caused any differences among bacterial orders, a generalized linear model was constructed using DESeq2 (Love, Huber & Anders, 2014). Significant differences were detected across treatments in *Vibrionales*, *Rhodobacterales*, *Alteromonadales*, *Cytophagales*, and *Burkholderiales* (Benjamini–Hochberg corrected *p* = 0.014, 0.045, 0.036, 0.011 and 0.007, respectively) taxa. To identify specific pair-wise differences in the relative abundance of these taxa across all the individual treatments (Control, Vcor alone, Hbv alone, Hbv + Vcor) we used Wald post hoc tests (Table 2).

As expected, addition of *V. coralliilyticus* resulted in increased *Vibrionales* affiliated sequence mean relative abundances in coral tissues (Fig. 4 purple lines). In the *V. coralliilyticus* treatment, the mean relative abundance of *Vibrionales* increased over 35% from the 4 to 8 h time point. Yet corals challenged with *V. coralliilyticus* in the presence of the predator *Halobacteriovorax* had an 84.74% reduction in *Vibrionales* compared to the *V. coralliilyticus* alone treatment. The combined treatment had a mean *Vibrionales* relative abundance of 1.43%, similar to the controls at 0.98%, while the *V. coralliilyticus* alone treatment had a mean 9.38% *Vibrionales* relative abundance across the whole experiment (Table 2; Fig. 4 box plots).

The *V. coralliilyticus* challenge also reported significantly higher relative abundance of two groups of taxa, *Cytophagales* and *Rhodobacterales*, compared to controls. Addition of *V. coralliilyticus* to corals increased the abundance of *Rhodobacterales* affiliated sequences in tissues to an even greater extent than *V. coralliilyticus* itself, and this increase persisted after *Vibrio* sequence abundances fell at later time points (Fig. 4 red lines). Similarly, patterns of increased *Rhodobacteraceae*-affiliated sequences (at the coral tissue loss disease front) in white syndrome affected corals have recently been reported (Pollock et al., 2017).

*Rhodobacterales* sequences increased throughout the experiment in the *V. coralliilyticus* addition treatment, nearly doubling at each time point starting from 6.99% (±0.05 SEM) relative abundance to a maximum mean value of 48.75% (± 0.14 SEM) in the final time point. However, during joint inoculation of *Halobacteriovorax* and *V. coralliilyticus*, *Rhodobacterales* showed no significant differences vs. controls. Similarly, there were no differences in the abundance of *Rhodobacterales* vs. controls in the treatment where *Halobacteriovorax* was added alone (Table 2). *Cytophagales* affiliated sequences also increased by several orders of magnitude, from <0.001% to 3.656%, early in the *V. coralliilyticus* addition experiment (Fig. 4 brown lines).

The two other taxa that significantly changed, but did so in different patterns, were *Burkholderiales* and *Alteromonadales* (Fig. 4 blue and green lines, respectively). The mean relative abundance for the order *Burkholderiales* was lowest (8.21%) in the *V.  coralliilyticus* treatment (Fig. 4 boxplots) and was significantly lower (*p* = 0.015) in the *V. coralliilyticus* versus the combined *Halobacteriovorax* and *V. coralliilyticus* treatment that had a relative abundance of 21.86% (Table 2; Fig. 4 box plots). *Alteromonadales* were 40.47% more abundant in the controls than the combined *Halobacteriovorax* and *V. coralliilyticus* treatment (*p* = 0.014) (Table 2; Fig. 4 box plots).

## Discussion

### Tracking microbial community dynamics after different bacterial challenges

High-throughput sequencing allows researchers to more easily document membership dynamics and community topology, yet we often lack the ability to confirm causal relationships among them. Manipulative studies are necessary to link cause and effect. While some host-microbe models can be more readily manipulated (e.g., mouse gut, squid light organ, and rhizosphere), there remain considerable methodological barriers for many systems, especially those for which gnotobiotic (germ-free) host animals are not available. Here we used individual and combinatorial bacterial challenges to a coral host in order to ask three specific questions: (1) How can inoculation of a foreign bacteria (*V. coralliilyticus*) alter the microbiome of a compromised host in a laboratory setting (2) Can predators of this taxa prevent or ameliorate the downstream effects of the alien challenges, and (3) Does the predator itself affect the coral microbiome?

*Vibrio coralliilyticus* is a known disease-causing pathogen of corals worldwide (Ben-haim, Zicherman-keren & Rosenberg, 2003; Wilson et al., 2013) and has been documented to induce bleaching and tissue loss in some species of corals (Ben-Haim et al., 1999; Ben-haim, Zicherman-keren & Rosenberg, 2003). Furthermore, experimental evidence has demonstrated that under increased thermal stress *V. coralliilyticus* concentrations rise dramatically in corals (Tout et al., 2015). However, the changes, if any, that *V. coralliilyticus* challenge causes to the microbial communities normally present in corals was previously unknown. *V. coralliilyticus* represents an alien or potential pathogen in this study, as it has not been previously shown to be a member of *Montastraea cavernosa* microbiome, and it remains unknown if it can infect certain Caribbean corals like *Montastraea cavernosa.* Never the less, determining how bacterial challenge can alter the normal flora of a host may provide insight into whether mutualists are lost and additional antagonisms arise during an infection cycle and thus contribute to secondary negative effects on animal hosts.

Here we show that a *V. coralliilyticus* inoculation not only changes its own relative abundance in the system (as would be expected) but also alters the microbiome in various ways, including increases in alpha and beta diversity (Fig. 3). However, when these corals were challenged with the *V. coralliilyticus* in the presence of the predator, these effects were diminished and resulted in almost no changes in the normal coral microbiome. Furthermore, addition of just the bacterial predator did not change the community in a similar fashion to the *V. coralliilyticus* challenge, suggesting that exposure to different foreign bacterial taxa (as opposed to any gram negative bacteria) will likely elicit variable downstream responses in the microbiome, unlike taxa that core members.

Addition of *V. coralliilyticus* led to an increase in relative abundance in a known group of opportunists of corals, the *Rhodobacterales* (Fig. 4). The increase in *Rhodobacterales* persisted at later time-points, even after the abundance of *V. coralliilyticus* had declined in the tissues. *Rhodobacterales* sequence abundances have been linked to disease outbreaks in white plague diseased *Siderastrea siderea* and *Diploria strigosa* corals (Cárdenas et al., 2012). Also, sequences from the family *Rhodobacteraceae* have been shown to increase by 4-folds at the lesion front of corals with white syndrome (Pollock et al., 2017). *Rhodobacterales* are fast growing taxa, capable of quickly responding to increasing availability of amino acids (Mayali et al., 2014), and could be responding to resources made available from cells damaged by *V. coralliilyticus.* Such a mechanism would explain associations between *Rhodobacterales* and many stressed or diseased corals. While the present study cannot distinguish whether these secondary *Vibrio*-induced *Rhodobacterales* are harmful to corals, the experimental framework used here could test this question in the future.

More broadly, *V. coralliilyticus* challenged samples showed a wider variety of bacteria sequences within the tissues (Fig. 3A). It is likely these opportunist species gained access and/or established within the tissue shortly after *Vibrio coralliilyticus* inoculation, as the increases in observed species persisted for the duration of the experiment (Table S2). However, the disproportionate impact of the *V. coralliilyticus* was not observed in samples challenged with the predator *Halobacteriovorax* suggesting that this is not a generalizable response to the wounding and addition of any kind of bacteria.

### *Halobacteriovorax* as a possible top down control of opportunists

We have previously cultivated *Halobacteriovorax* from multiple-species of corals, and used long-term microbial time series data to show that, despite its low abundance, it is a core member of the microbiome of several coral genera (Welsh et al., 2016). Here we used bacterial challenge experiments to demonstrate that *Halobacteriovorax* can protect its coral host by consuming its prey *V. coralliilyticus*. We found that the application of *Halobacteriovorax* at the same time as *V. coralliilyticus* can prevent detectable changes in the relative abundance of *V. coralliilyticus* in *M. cavernosa* coral tissue after challenge. The *Halobacteriovorax* alone treatment showed higher variance in the mean relative abundance of *Vibrionales* than the controls or combined treatment (Fig. 4), but the mean was not significantly different than the controls. Co-inoculations of this predator with *V. coralliilyticus* showed no significant differences in the abundance of *Vibrionales* in coral tissues versus control inoculations at any time in the course of the experiment (Fig. 4 purple lines). Thus it is likely that these predators consumed the *Vibrio* immediately or at the point of inoculation, and therefore provided a biotic barrier to the host tissues. The ability of *Halobacteriovorax* to mitigate inoculation, if added hours or days after a *V. coralliilyticus* challenge, remains unknown. At the same time the generality of this effect of the *Vibrio* and the predatory *Halobacteriovorax* remains untested, but could be evaluated using similar methods to those we describe here. For example, additional types of pathogens that show clear infection signs upon addition or the use of more commensal strains of bacteria as controls would strengthen support for this hypothesis.

Phage have already been shown to be effective against *V. coralliilyticus* (Cohen et al., 2013), and likely play a role in controlling natural populations of *V. coralliilyticus* in the environment, which is similar to what has been suggested for phage and *V. cholerae* (Faruque et al., 2005). Phages also provide an antimicrobial function in the mucus layer of corals (Barr et al., 2013; Barr et al., 2015) and are often considered the main top-down control mechanism of bacteria in some systems. However in certain circumstances, *Halobacteriovorax* predation has been shown to be a more dominant factor in bacterial mortality than viral lysis (Williams et al., 2015). In addition, predatory bacteria are thought to play a major role in controlling pathogenic *Vibrio* in seawater and shellfish (Richards et al., 2012). In our study we show predatory *Halobacteriovorax sp.* PA1 is effective against *V. coralliilyticus* BAA 450 and other *Vibrio* strains, offering further support to the hypothesis that bacterial predators are likely to play a role in controlling populations in the environment. In a similar fashion to phages, *Halobacteriovorax* thus mediates top-down control of pathogens by preventing initial invasion of the host.

### Microbiome manipulation validates previous network analysis   predictions

A small but growing body of research suggests *Halobacteriovorax* naturally occur and regularly interact with members of the coral microbiome. For example, a previous metagenomic study of *P. astreoides* from Panama reported that sequences similar to predatory *Halobacteriovorax* were among the most commonly identified bacterial annotations in the coral microbiome (Wegley et al., 2007). Furthermore, we found *Halobacteriovorax* was present in ∼80% of samples collected approximately monthly from three genera of Caribbean corals across a three-year time span. Network analysis of 198 of these samples detected intriguing co-occurrences between these predators and other taxa (Welsh et al., 2016). Here in the bacterial challenge study, we validated several of the co-occurrence patterns detected in our network analysis. For example, in our networks from *Agaricia* corals, *Bdellovibrionales* (the order of *Halobacteriovorax*) positively co-occurred with both *Vibrionales* and *Cytophagales* in the field (Welsh et al., 2016). Here we experimentally demonstrated that *Halobacteriovorax* directly alters the abundance of both of these taxa. We show here that a *V. coralliilyticus* challenge is associated with significant increases in *Cytophagales* relative abundance *in vivo* as well, suggesting there is a more direct interaction between these two taxa (Table 2). This work lends support to the use of networks to provide a predictive understanding of the microbiome’s function and dynamics in natural systems

### Caveats and considerations

While this limited laboratory study suggests that *Vibrio coralliilyticus* alters the microbiome of coral tissue, whether these downstream responses of the microbiome are the result of wounding and challenge with any putative pathogen remains untested. At the same time, both the absolute numbers of *V. coralliilyticus* added in this experiment were not environmentally relevant and whether *V. coralliilyticus is a pathogen of M. cavernosa* is still unknown, shedding doubt on whether these same effects would be seen in true infection scenarios. Yet the patterns of change in the community after addition of *V. coralliilyticus* remarkably mirror the changes we found in corals exposed to stressors in the environment. For example, we found that overall beta-diversity and the relative abundance of opportunists such as *Rhodobacterales* increase in corals under threat (Zaneveld et al., 2016). This striking similarity of these disparate works can be interpreted in two ways: (1) any stress to a system, whether it be a direct bacterial challenge or exposure to imperfect conditions on a reef, drive these changes in the microbiome or that (2) specific infection by groups of foreign bacteria are responsible for such affects. To distinguish these alternatives, more comprehensive experiments should be conducted such as those using a variety of host species and putative pathogens, additional commensals for controls, challenge trials that have different dilutions and more environmentally relevant concentrations of bacterial inoculate, and evaluations of the changes in the bacterial community in terms of finer taxonomic resolution as well as absolute abundances (as opposed to relative abundance) measures.

## Conclusions

Corals and other marine organisms are in constant contact with an array of distinct microbes. In the face of this constant microbial challenge healthy host microbiomes are robust to change unless challenged with foreign agents or poor environmental conditions. In this laboratory system, a challenge by *V. coralliilyticus* resulted in a destabilized host microbiome in which opportunists bloomed, potentially further exacerbating the negative effects of the initial inoculation. Recent evidence also supports the hypothesis that commensal or mutualistic host-associated microbes offer protection against invasive pathogens either by depriving these alien bacteria of essential nutrients or acting as a physical barrier to host attachment (Weyrich et al., 2014). Here we show additional evidence by which host-associated predatory bacteria protect the host microbiome through direct consumption of prey. The ability to manipulate the microbiome and therefore test various hypotheses about the principles that govern microbial community assembly, dynamics, and functions, especially in terms of how these relate to host health, remain a challenge for our field (Waldor et al., 2015). As our ability to culture and test the effects of more coral microbial taxa improves, so will our methods to manipulate and track the dynamics of the microbiome in real time and under more realistic environmental conditions. Such efforts will allow us to gain a better understanding of the relationships among members of the microbiota and between the microbiome with the host, and which ideally will result in better understanding and management of bacterial mediated diseases.

##  Supplemental Information

10.7717/peerj.3315/supp-1 Table S1 Table S1 Estimation of the actual number of cells transferred using the swab transfer method. Direct cell counts using epifluorescent microscopy to quantify the number of cells transferred using the swab pellet transfer method.

10.7717/peerj.3315/supp-2 Table S2 Alpha diversity by bacterial challenge treatment at each time point Alpha diversity at a rarefaction sequence depth of 11716 reads from coral samples in microbiome manipulation experiment.

10.7717/peerj.3315/supp-3 Supplemental Information 3 OTU mapping file

10.7717/peerj.3315/supp-4 Supplemental Information 4 OTU table for deseq2

10.7717/peerj.3315/supp-5 Supplemental Information 5 Main OTU table

10.7717/peerj.3315/supp-6 Supplemental Information 6 Tree file

10.7717/peerj.3315/supp-7 Supplemental Information 7 Code

## Additional Information and Declarations

### Field Study Permissions

The following information was supplied relating to field study approvals (i.e., approving body and any reference numbers): The Florida Keys National Marine Sanctuary and NOAA coral nursery in Key West approved permits (#FKNMS-2010-123) and provided coral specimen for this work.

### Data Availability

The following information was supplied regarding data availability: The mapping file, biom otu table, source code and tree file can be found in the Supplemental Information.
