# Supplementary material for: Alien vs. predator: bacterial challenge alters coral microbiomes unless controlled by Halobacteriovorax predators
Source: PeerJ. 2017 May 31;5:e3315. doi: 10.7717/peerj.3315 (PMC5455293; doi:10.7717/peerj.3315)
Supplement: Table S1 — Estimation of the actual number of cells transferred using the swab transfer method. Direct cell counts using epifluorescent microscopy to quantify the number of cells transferred using the swab pellet transfer method. [file peerj-05-3315-s001.docx]

**Supplemental Tables**

Table S1:

Estimation of the actual number of cells transferred using the swab transfer method. Direct cell counts using epifluorescent microscopy quantify the number of cells transferred using the swab pellet transfer method.

|  | **Bacterial Challenge Swab Transfer Estimation** | |
| --- | --- | --- |
|  | **Cells Before Swab Pellet Transfer** | **Total Cell Transferred** |
| ***Replicate 1*** | **2.25E+09** | **5.09E+08** |
| ***Replicate 1*** | **3.92E+09** | **6.56E+08** |
| ***Replicate 1*** | **5.90E+09** | **6.42E+08** |
| ***Average*** | **4.02E+09** | **6.02E+08** |
| ***Standard Deviation*** | **1.82E+09** | **8.12E+07** |
