# Supplementary material for: Alien vs. predator: bacterial challenge alters coral microbiomes unless controlled by Halobacteriovorax predators
Source: PeerJ. 2017 May 31;5:e3315. doi: 10.7717/peerj.3315 (PMC5455293; doi:10.7717/peerj.3315)
Supplement: Table S2 — Alpha diversity at a rarefaction sequence depth of 11716 reads from coral samples in microbiome manipulation experiment. [file peerj-05-3315-s002.docx]

**Supplemental Tables**

Table S2. Alpha diversity altered by bacterial challenge treatment at each time point. Alpha diversity at a rarefaction sequence depth of 11716 reads from sequences derived from coral samples in microbiome manipulation experiment and reported for each time point.

|  | **Bacterial Challenge Treatment** | | | |
| --- | --- | --- | --- | --- |
| **Time** | **Control** | **Hbv** | **V. cor** | **Hbv+V. cor** |
| ***4*** | **171.7 ± 13.3** | **208.7 ± 61.5** | **212.2 ± 38.7** | **161.9 ± 43.7** |
| ***8*** | **164.6 ± 25.4** | **201.0 ± 86.6** | **260.0 ± 62.7** | **175.2 ± 22.5** |
| ***24*** | **200.8 ± 60.4** | **176.7 ± 23.4** | **249.6 ± 127.4** | **130.1 ± 4.5** |
| ***32*** | **177.6 ± 62.3** | **159.6 ± 26.4** | **313.4 ± 19.6** | **191.7 ± 36.1** |
