# Supplementary material for: Alien vs. predator: bacterial challenge alters coral microbiomes unless controlled by Halobacteriovorax predators
Source: PeerJ. 2017 May 31;5:e3315. doi: 10.7717/peerj.3315 (PMC5455293; doi:10.7717/peerj.3315)
Supplement: Supplemental Information 7 [file peerj-05-3315-s007.rtf]

# Data notebook for M. cavernosa 16S rRNA V4 reads#### MacQIIME code start ##### To view summary statistics of the OTU table, run:biom summarize-table -i m_cavernosa_otu_table.biom -o otu_table_summary.txt### Alpha Diversity of all samples alpha_rarefaction.py -i m_cavernosa_otu_table.biom -m m_cavernosa_mapping.txt -o mcav_arare -p alpha_params.txt -t otus/rep_set.tre### Alpha Diversity at each timepoint alpha_rarefaction.py -i m_cavernosa_otu_table_t4.biom -m m_cavernosa_mapping.txt -o t4_arare -p alpha_params.txt -t otus/rep_set.trealpha_rarefaction.py -i m_cavernosa_otu_table_t8.biom -m m_cavernosa_mapping.txt -o t8_arare -p alpha_params.txt -t otus/rep_set.trealpha_rarefaction.py -i m_cavernosa_otu_table_t24.biom -m m_cavernosa_mapping.txt -o t24_arare -p alpha_params.txt -t otus/rep_set.trealpha_rarefaction.py -i m_cavernosa_otu_table_t32.biom -m m_cavernosa_mapping.txt -o t32_arare -p alpha_params.txt -t otus/rep_set.tre# This script compares the alpha diversity between groups of samples found in a collated alpha diversity filecompare_alpha_diversity.py -i chao1.txt -m m_cavernosa_mapping.txt -c treatment -d 11716 -o Treatment_chao1compare_alpha_diversity.py -i observed_species.txt -m m_cavernosa_mapping.txt -c treatment -d 11716 -o Treatment_obs_spcompare_alpha_diversity.py -i equitability.txt -m m_cavernosa_mapping.txt -c treatment -d 11716 -o Treatment_equitacompare_alpha_diversity.py -i observed_species.txt -m m_cavernosa_mapping.txt -c time_point -d 11716 -o Time_point_obs_spcompare_alpha_diversity.py -i observed_species.txt -m m_cavernosa_mapping.txt -c tank -d 11716 -o tank_obs_sp### Beta Diversity of all samples beta_diversity_through_plots.py -i m_cavernosa_otu_table.biom -m m_cavernosa_mapping.txt -o bdiv_no_t0_even11716 -t otus/rep_set.tre -e 11716# This script compares the beta diversity between groups of samples make_distance_boxplots.py -m m_cavernosa_mapping.txt -d bdiv_no_t0_even11716/weighted_unifrac_dm.txt -f treatment -n 999 -o weighted_boxplots_11716_supress --suppress_all_within --suppress_all_between --suppress_individual_between --save_raw_datamake_distance_boxplots.py -m m_cavernosa_mapping.txt -d bdiv_no_t0_even11716/unweighted_unifrac_dm.txt -f treatment -n 999 -o unweighted_boxplots_11716_final --suppress_all_within --suppress_all_between --suppress_individual_between --save_raw_datamake_distance_boxplots.py -m m_cavernosa_mapping.txt -d bdiv_no_t0_even11716/weighted_unifrac_dm.txt -f time_point -n 10 -o weighted_boxplots_11716_time --save_raw_datamake_distance_boxplots.py -m m_cavernosa_mapping.txt -d bdiv_no_t0_even11716/weighted_unifrac_dm.txt -f tank -n 10 -o weighted_boxplots_11716_tank --save_raw_data#### MacQIIME code end ######## R code start #### rm(list=ls())library("phyloseq"); packageVersion("phyloseq")library("DESeq2")packageVersion("DESeq2")# Use Phyloseq import function for QIIME databiom <- import_biom("deseq_otu_table_filtered_order_level.biom", taxaPrefix = "greengenes")map_file <- import_qiime_sample_data("m_cavernosa_mapping.txt")full_data <- merge_phyloseq(biom, map_file) ##### specify the model you want to run based on the mapping filedds <- phyloseq_to_deseq2(full_data, ~time_point+treatment+time_point:treatment)#### Now I use the Likelihood ratio test for GLMs to test treatment and timedds_lrt_time_point <- DESeq(dds,test='LRT', reduced=~treatment)results(dds_lrt_time_point)res_lrt_time_point <- results(dds_lrt_time_point)res_lrt_time_point_ordered <- res_lrt_time_point[order(res_lrt_time_point$padj),]write.csv(as.data.frame(res_lrt_time_point_ordered),          file="res_time_treatment_default_ordered_all_time_pointL4_filt.csv")dds_lrt_treatment <- DESeq(dds,test='LRT', reduced=~time_point)results(dds_lrt_treatment)res_lrt_treatment <- results(dds_lrt_treatment)res_lrt_treatment_ordered <- res_lrt_treatment[order(res_lrt_treatment$padj),]write.csv(as.data.frame(res_lrt_treatment_ordered),          file="res_time_treatment_default_ordered_all_treatment_L4_filt.csv")dds_lrt_treatment_time <- DESeq(dds,test='LRT', reduced=~time_point+treatment)results(dds_lrt_treatment_time)res_lrt_treatment_time <- results(dds_lrt_treatment_time)res_lrt_treatment_time_ordered <- res_lrt_treatment_time[order(res_lrt_treatment_time$padj),]write.csv(as.data.frame(res_lrt_treatment_time_ordered),          file="res_time_treatment_default_ordered_all_treatment_timeL4_filt.csv")dds_wald <- DESeq(dds)res_time_treatment_default <- results(dds_wald, contrast = c('treatment', 'control', 'vc'))head(res_time_treatment_default)summary(res_time_treatment_default)res_time_treatment_default_ordered <- res_time_treatment_default[order(res_time_treatment_default$pvalue),]head(res_time_treatment_default_ordered)head(res_time_treatment_default_ordered, n=10)write.csv(as.data.frame(res_time_treatment_default_ordered),          file="res_time_treatment_default_ordered_L4_filt_con_vc.csv")resultsNames(dds_wald)res_wald_treatment_default <- results(dds_wald, contrast = c('treatment', 'control', 'vc_and_hbv'))head(res_wald_treatment_default)summary(res_wald_treatment_default)res_wald_treatment_default_ordered <- res_wald_treatment_default[order(res_wald_treatment_default$pvalue),]head(res_wald_treatment_default_ordered)head(res_wald_treatment_default_ordered, n=10)write.csv(as.data.frame(res_wald_treatment_default_ordered),          file="res_time_treatment_default_ordered_L4_filt_con_vc_and_hbv.csv")resultsNames(dds_wald)res_wald_treatment_c_bx_default <- results(dds_wald, contrast = c('treatment', 'control', 'hbv'))head(res_wald_treatment_c_bx_default)summary(res_wald_treatment_c_bx_default)res_wald_treatment_c_bx_default_ordered <- res_wald_treatment_c_bx_default[order(res_wald_treatment_c_bx_default$pvalue),]head(res_wald_treatment_c_bx_default_ordered)head(res_wald_treatment_c_bx_default_ordered, n=10)write.csv(as.data.frame(res_wald_treatment_c_bx_default_ordered),          file="res_time_treatment_default_ordered_L4_filt_con_hbv.csv")res_wald_treatment_vc_vcbx_default <- results(dds_wald, contrast = c('treatment', 'vc_and_hbv', 'vc'))head(res_wald_treatment_vc_vcbx_default)summary(res_wald_treatment_vc_vcbx_default)res_wald_treatment_vc_vcbx_default_ordered <- res_wald_treatment_vc_vcbx_default[order(res_wald_treatment_vc_vcbx_default$pvalue),]head(res_wald_treatment_vc_vcbx_default_ordered)head(res_wald_treatment_vc_vcbx_default_ordered, n=10)write.csv(as.data.frame(res_wald_treatment_vc_vcbx_default_ordered),          file="res_time_treatment_default_ordered_L4_filt_vc_vchbv.csv")res_wald_treatment_hbv_vcbx_default <- results(dds_wald, contrast = c('treatment', 'vc_and_hbv', 'hbv'))head(res_wald_treatment_hbv_vcbx_default)summary(res_wald_treatment_hbv_vcbx_default)res_wald_treatment_hbv_vcbx_default_ordered <- res_wald_treatment_hbv_vcbx_default[order(res_wald_treatment_hbv_vcbx_default$pvalue),]head(res_wald_treatment_hbv_vcbx_default_ordered)head(res_wald_treatment_hbv_vcbx_default_ordered, n=10)write.csv(as.data.frame(res_wald_treatment_hbv_vcbx_default_ordered),          file="res_time_treatment_default_ordered_L4_filt_hbv_vchbv.csv")res_wald_treatment_hbv_vc_default <- results(dds_wald, contrast = c('treatment', 'vc', 'hbv'))head(res_wald_treatment_hbv_vc_default)summary(res_wald_treatment_hbv_vc_default)res_wald_treatment_hbv_vc_default_ordered <- res_wald_treatment_hbv_vc_default[order(res_wald_treatment_hbv_vc_default$pvalue),]head(res_wald_treatment_hbv_vc_default_ordered)head(res_wald_treatment_hbv_vc_default_ordered, n=10)write.csv(as.data.frame(res_wald_treatment_hbv_vc_default_ordered),          file="res_time_treatment_default_ordered_L4_filt_hbv_vc.csv")
